# Interleukin-13 maintains the stemness of conjunctival epithelial cell cultures prepared from human limbal explants

**DOI:** 10.1371/journal.pone.0211861

**Published:** 2019-02-11

**Authors:** Andrea Stadnikova, Peter Trosan, Pavlina Skalicka, Tor Paaske Utheim, Katerina Jirsova

**Affiliations:** 1 Laboratory of the Biology and Pathology of the Eye, Institute of Biology and Medical Genetics, First Faculty of Medicine, Charles University and General University Hospital in Prague, Prague, Czech Republic; 2 Department of Ophthalmology, First Faculty of Medicine, Charles University and General University Hospital in Prague, Prague, Czech Republic; 3 Department of Medical Biochemistry, Oslo University Hospital, Oslo, Norway; 4 Department of Plastic and Reconstructive Surgery, Oslo University Hospital, Oslo, Norway; Cedars-Sinai Medical Center, UNITED STATES

## Abstract

To use human limbal explants as an alternative source for generating conjunctival epithelium and to determine the effect of interleukin-13 (IL-13) on goblet cell number, mucin expression, and stemness. Human limbal explants prepared from 17 corneoscleral rims were cultured with or without IL-13 (IL-13+ and IL-13-, respectively) and followed up to passage 2 (primary culture [P0]–P2). Cells were characterized by alcian blue/periodic acid–Schiff (AB/PAS) staining (goblet cells); immunofluorescent staining for p63α (progenitor cells), Ki-67 (proliferation), MUC5AC (mucin, goblet cells), and keratin 7 (K7, conjunctival epithelial and goblet cells); and by quantitative real-time polymerase chain reaction for expression of the p63α (*TP63*), *MUC5AC*, *MUC4* (conjunctival mucins), *K3*, *K12* (corneal epithelial cells), and *K7* genes. Clonogenic ability was determined by colony-forming efficiency (CFE) assay. Using limbal explants, we generated epithelium with conjunctival phenotype and high viability in P0, P1, and P2 cultures under IL-13+ and IL-13- conditions, i.e., epithelium with strong K7 positivity, high *K7* and *MUC4* expression and the presence of goblet cells (AB/PAS and MUC5AC positivity; *MUC5AC* expression). p63α positivity was similar in IL-13+ and IL-13- cultures and was decreased in P2 cultures; however, there was increased *TP63* expression in the presence of IL-13 (especially in the P1 cultures). Similarly, IL-13 increased proliferative activity in P1 cultures and significantly promoted P0 and P1 culture CFE. IL-13 did not increase goblet cell number in the P0–P2 cultures, nor did it influence *MUC5AC* and *MUC4* expression. By harvesting unattached cells on day 1 of P1 we obtained goblet cell rich subpopulation showing AB/PAS, MUC5AC, and K7 positivity, but with no growth potential. In conclusion, limbal explants were successfully used to develop conjunctival epithelium with the presence of putative stem and goblet cells and with the ability to preserve the stemness of P0 and P1 cultures under IL-13 influence.

## Introduction

The conjunctiva is composed of a non-keratinizing stratified epithelium with interspersed goblet cells (GCs) and a vascularized stroma. It contributes to the integrity of the ocular surface by producing the mucin component of the tear film, forming a mechanical barrier against pathogens and being a part of the mucosal immune defense system [1–4]. Mucins are high–molecular weight glycoproteins that lubricate the ocular surface and stabilize the ocular film. Human GCs secrete the gel-forming mucin MUC5AC, soluble MUC2, and membrane-associated MUC16. Corneal and conjunctival epithelial cells express the membrane-associated MUC1 and MUC16, while MUC4 is prevalently expressed by conjunctival cells [3, 5].

Corneal epithelium is maintained by limbal stem cells located in palisades of Vogt [6]. Conjunctival stem cells are bipotential and give rise to both epithelial cells and GCs [7]. Stem cells are distributed throughout the conjunctival tissue, with density being highest in the nasal part of the lower fornix and the medial canthus [8, 9], where GC density is also the highest [2]. Differentiation into GCs occurs later during the stem cell life cycle at the stage of transient amplifying cell [7]. GCs can be generated also from limbal epithelial cells influenced by the conjunctival environment [10].

The effect of interleukin-13 (IL-13), a T helper 2-type cytokine [11], on GCs and mucus production in healthy and diseased tissues has been intensively studied in other tissues, for example airway epithelium [12]. In conjunctiva, increase of IL-13 is believed to be involved in the pathogenesis of conjunctival immune diseases involving stimulation of GC numbers, mucus production and fibroblasts proliferation (atopic and vernal keratoconjunctivitis, giant papillary conjunctivitis, mucous membrane pemhigoid) [13–16]. Moreover, it appears that its presence in healthy conjunctival tissue is necessary for GC differentiation and homeostasis [17]. In epidermal tissue, IL-13 could be important for protection against environmental stressors and carcinogenesis [18]. So far, only a few studies have focused on IL-13 and conjunctival tissue prepared *in vitro* [19–22]. In *in vitro* murine experiments, IL-13 stimulated conjunctival GC proliferation [19–21]; however, its effect on MUC5AC is inconsistent; one study showed it had no effect on MUC5AC secretion [20], and another reported a stimulatory effect [19]. The addition of IL-13 to human conjunctival epithelial cell cultures stimulated MUC5AC secretion [22]; however, its effect on GC numbers or *MUC5AC* expression in human conjunctival tissue prepared *in vitro* has not been studied so far.

Ocular surface deterioration associated with dry eye, conjunctival damage, and scarring is usually accompanied by decreased or even absent GCs and mucin (for review see [3, 23]). Most diseases or conditions affecting the ocular surface are related to the destruction of both the corneal and conjunctival epithelium, i.e., reconstruction in such cases requires the regeneration of both tissues [24]. Experiments on the development of human tissue–engineered conjunctival equivalents have been underway for almost 30 years [25, 26]; the search for convenient cultivation conditions continues because engineering full-fledged conjunctival tissue from two cell types is much more complicated in comparison to that for corneal epithelium composed of only epithelial cells. Thus, so far experimental studies using cultured conjunctival epithelial cells for conjunctival reconstruction prevail [26, 27] and studies in patients are very rare [28]. However, the rationale for improving the culture protocols for human conjunctival epithelial cell sheets is substantiated by their ability to treat limbal stem cell deficiency (LSCD) without the need for immunosuppression in cases where autologous limbal tissue is not available (e.g. bilateral total LSCD) [24, 29–31].

Following the landmark publication by Rama *et al*. published in 2010 [32] demonstrating that the percentage of a marker for undifferentiated cells in transplanted sheets is linked to success following transplantations in patients with LSCD, research on how to support the maintenance of stemness in cultured sheets intended for ocular surface reconstruction has significantly gained momentum. In light of this, we herein explored to what extent IL-13 could influence the stemness of cultured conjunctival cells. In addition, we tested whether explant culture from human corneoscleral rims could be used for engineering conjunctival epithelium and evaluated the influence of human recombinant IL-13 on GC numbers and mucin expression in the conjunctival cultures.

## Materials and methods

### Tissue specimens

The study followed the standards of the Ethics Committees of the General Teaching Hospital and the First Faculty of Medicine of Charles University, Prague, Czech Republic (Ethics Committee approval no. 8/14 held on January 23, 2014), and adhered to the tenets set out in the Declaration of Helsinki. We obtained human cadaver corneoscleral rims from 17 donors, which were surplus from surgery and stored in Eusol-C (Alchimia, Padova, Italy), from the Department of Ophthalmology, General University Hospital in Prague, Czech Republic, for the study. On the use of the corneoscleral rims, based on Czech legislation on specific health services (Law Act No. 372/2011 Coll.), informed consent is not required if the presented data are anonymized in the form. The mean donor age ± standard deviation (SD) was 63.5 ± 6.5 years. The tissue was collected within 24 h from death. After the surgery, the corneoscleral rims were stored in Eusol-C at 4°C until limbal explants were prepared for cultivation. The mean storage time ± SD (from tissue collection until explantation) was 6.2 ± 3.2 days. Conjunctival impression cytology was performed on two healthy adult volunteers who had provided informed consent (EK-2370/14 S-IV approved on 12/11/2014). Conjunctival tissue with pterygium was harvested from two patients at the Department of Ophthalmology, General University Hospital in Prague, Czech Republic. Patients provided informed consent before the pterygium removal surgery (EK-1570/11 S-IV approved on 10/13/2011).

### Limbal explants and cell culture

The surplus cornea and sclera of the corneoscleral rims were cut off, and then the limbal portion, including the residual conjunctival tissue, was cut into 12 pieces (approximately 2 × 3 mm) and placed corneoconjunctival epithelial side down in a 24-well plate (TPP Techno Plastic Products AG, Trasadingen, Switzerland). Six explants were placed directly on the plastic bottom of the plate and six pieces were placed on Thermanox plastic cell culture coverslips (Nunc, Thermo Fisher Scientific, Rochester, NY, USA) for histological or immunohistochemical staining of cultured cells. Each explant was covered with one drop (35 μl) of complete medium and maintained at 37°C in 5% CO_2_. Complete medium was changed every day until cell outgrowth was seen. Then, the tissue was covered with 1 ml complete medium, which was changed three times a week until the cells were 90–100% confluent.

The complete medium consisted of Dulbecco’s modified Eagle medium mixed 1:1 with Ham’s F12 (DMEM/F12 with GlutaMAX) and supplemented with 10% fetal bovine serum, 1.5 g/l sodium bicarbonate (7.5% solution), 10 mM HEPES, 1 μg/ml insulin–transferrin–selenium, 1% Antibiotic-Antimycotic solution (100 IU/ml penicillin, 100 μg/ml streptomycin, 0.25 μg/ml fungizone), and 10 ng/ml recombinant human epidermal growth factor (all, Gibco, Thermo Fisher Scientific, Paisley, UK). Half of the donor explants were cultured in complete medium supplemented with 10 ng/ml recombinant human IL-13 (BioLegend, San Diego, CA, USA). Similarly, after passage, the cells were cultured in complete medium with (IL-13+) or without IL-13 (IL-13-). At day 1 of passage 1 (P1d1) cultivation, numerous cells with prevalent GC morphology that were only slightly attached and unattached were found on top of the firmly attached epithelial cells. The unattached cells were harvested on P1d1 and designated the P1d1 subpopulation. When primary (P0) cultures and P1 cultures were 90–100% confluent and when P2 cultures had been cultured for 12–14 days from the beginning of culture, the cells were detached using TrypLE Express (Gibco, Thermo Fisher Scientific). P0 and P1 cells intended for cell passaging were seeded in 24-well plates at concentrations of 1.5 × 10^4^ cells per well. Experiments were run on P0, P1, and P2 cells, and partially on the P1d1 fraction. All experiments were repeated at least three times.

### 3T3 mouse fibroblast feeder layer

3T3 mouse fibroblasts were cultivated in DMEM supplemented with 10% newborn calf serum and 1% Antibiotic-Antimycotic solution (all, Gibco, Thermo Fisher Scientific). The cultures were maintained at 37°C and 5% CO_2_ and passaged after reaching 90% confluence. The mouse fibroblasts were growth-arrested by 2-h incubation with 12 μg/ml Mitomycin-C Kyowa (NORDIC Pharma, Prague, Czech Republic) and plated on a 6-well plate at a density of 2.6 × 10^5^ cells per well.

### Cell viability

Cell viability was assessed by staining with 0.4% trypan blue (Gibco, Thermo Fisher Scientific). Unstained live cells and stained dead cells were counted with a hemocytometer under an inverted phase contrast microscope. Cell viability was calculated as follows: viability (%) = live cells/(live + dead cells) × 100.

### Colony-forming efficiency (CFE) assay

Cultured cells were detached, centrifuged, single-cell suspended, and plated at a density of 700 or 1000 viable cells per well in 6-well plates containing growth-arrested 3T3 mouse fibroblasts. Similarly, to determine the clonal growth ability of GCs one day after passage, the unattached GC-enriched subpopulations were harvested and plated at a density of 1000 or 2000 viable cells per well. All experiments were performed in triplicate for each donor and condition (i.e., IL-13- and IL-13+). On day 12 or 13 after the cultures were started, the colonies were washed with phosphate-buffered saline (PBS; Gibco, Thermo Fisher Scientific), fixed with ice-cold methanol for 30 min at -20°C, rehydrated in PBS, and stained for 5 min at 37°C with 2% rhodamine B (Merck KGaA, Darmstadt, Germany). After the rhodamine B had been removed, the colonies were washed with tap water until optimal staining intensity was achieved. The plates were photographed, and the total number of colonies was counted using NIS Elements software (Laboratory Imaging, Prague, Czech Republic). The total CFE (%) was calculated as follows: (total number of colonies formed at the end of growth period/total number of viable cells seeded) × 100.

### Cytospin

Superfrost Plus slides (Thermo Fisher Scientific, Braunschweig, Germany) and filter paper were placed in the sample chamber of a cytospin centrifuge. Cell aliquots (100 μl) were loaded into each chamber and centrifuged at 180 ×*g* for 8 min. After centrifugation, the slides were air-dried and stored at -20°C until used.

### Alcian blue/periodic acid–Schiff (AB/PAS) staining

At the end of the culture, cells grown on Thermanox coverslips were washed with PBS and fixed (solution of 2 ml glacial acetic acid, 1.9 ml 40% formaldehyde, 29.5 ml 95% ethanol, 10.5 ml distilled water) for 10 min and stored in 70% ethanol at 4°C until used. Cells centrifuged on Superfrost Plus slides were taken out of the -20°C freezer and after 30 min on air fixed and AB/PAS stained. AB/PAS staining for demonstrating GC positivity was performed as follows: Tap water was used to hydrate fixed cells and to wash cells after each step of staining. Cells were stained with alcian blue (pH 2.5, Sigma-Aldrich, St. Louis, MO, USA) for 15 min, treated with freshly prepared 0.5% periodic acid solution for 10 min, stained for 6 min with Schiff’s reagent (Sigma-Aldrich; diluted 1:3 in distilled water) and counterstained with hematoxylin solution modified according to Gill III (Merck KGaA) for 5 sec. At the end, the cells were washed with Scott’s water (0.2 g sodium bicarbonate, 1 g magnesium sulfate, 100 ml distilled water), air-dried and mounted in Aquatex medium (Merck KGaA). The staining results were as follows: acidic mucins were stained blue, neutral mucins were stained magenta, and acidic and neutral mucins were stained purple [33].

All images were acquired with an Olympus BX51 microscope (Tokyo, Japan), a ProgRes C12 plus camera (Jenoptik Laser, Optik, Systeme GmbH, Jena, Germany), and NIS Elements software (Laboratory Imaging). Specimens harvested by bulbar conjunctival impression cytology and cryosectioned pterygium specimens were used as a positive control for AB/PAS staining. The absolute number of AB/PAS-positive GCs per image area (0.58 mm^2^) was counted on 10 randomly captured images per well from at least five independent tissue donors, and the final results are expressed as absolute cell numbers per 1 mm^2^. The percentage of AB/PAS-positive GCs in P1d1 population was counted on at least 500 cells/ donor (range 500–2200 cells/ donor).

### Impression cytology

Upper bulbar conjunctival impression cytology specimens were obtained from living healthy donors after the application of 0.4% oxybuprocaine hydrochloride eye drops as topical anesthesia [34]. For immunofluorescent staining, cells were harvested using sterile, single-pack Millicell inserts (PICM01250, Millipore, Bedford, MA, USA). The inserts with the impressed cells were stored at -80°C until analysis. For AB/PAS staining, nitro acetate cellulose filter papers (GSWP 0.4700, 0.22-μm pore size, Millipore) were used for cell harvesting.

### Indirect fluorescent immunocytochemistry

The cells that had been cultured on Thermanox slides or centrifuged on Superfrost Plus slides were washed twice with PBS and, according to the primary antibody to be used, fixed in 4% paraformaldehyde for 10 min at room temperature (for keratin 7 [K7], a conjunctival cell keratin [3, 34]; Ki-67, a proliferation marker of a nuclear protein expressed in all phases of the active cell cycle [35]; and p63α [the p63α isoform encoded by the tumor protein P63 gene *TP63*], a p53-related nuclear protein included in the regulation of epithelial cell differentiation and proliferation [36] and considered a putative marker of limbal epithelial stem cells and young transient amplifying cells [37, 38]) or in ice-cold methanol for 5 min at -20°C (for MUC5AC, a GC-specific mucin [3]), and thereafter hydrated in PBS. Table 1 lists the details of all primary antibodies. The negative control did not contain primary antibody.

**Table 1:** Primary antibodies.

Target protein	Animal	Clone	Manufacturer	Catalog Number	Original concentration	Dilution
K7	mouse	OV-TL 12/30	DAKO Cytomation, Glostrup, Denmark	M7018	243 mg/L	1:50
Ki-67	mouse	MIB-1	DAKO Cytomation, Glostrup, Denmark	M7240	80 mg/L	1:50
p63α	rabbit	Polyclonal	Cell Signalling Technology, Danvers, MA, USA	4892	17 μg/ml	1:50
MUC5AC	rabbit	Polyclonal	Thermo Fisher Scientific, Rockford, IL, USA	PA5-34612	1 mg/ml	1:400

#### K7 staining and Ki-67 and p63α double staining

After hydration in PBS and washing three times in 0.5% Tween 20 (Sigma-Aldrich) and three times in PBS, cells were blocked for 1 h in 5% goat serum and 0.3% Triton X-100 (Sigma-Aldrich) diluted in PBS, followed by 1-h incubation at room temperature with primary antibody diluted in 0.1% bovine serum albumin (K7) or blocking solution (Ki-67 and p63α). Then, the cells were rinsed three times in 0.5% Tween 20 and incubated for 1 h at room temperature with the respective secondary antibody diluted in 0.1% bovine serum albumin (K7) or blocking solution (Ki-67 and p63α): goat anti-mouse Alexa Fluor 488 (1:500, A11029), goat anti-mouse Alexa Fluor 594 (1:500, A11032), and goat anti-rabbit Alexa Fluor 488 (1:500, A11034, all secondary antibodies were from Life Technologies, Eugene, OR, USA). After rinsing three times in 0.5% Tween 20, followed by rinsing in PBS, the cells were mounted with VectaShield-DAPI (4′ 6-diamidino-2-phenylindole) (Vector Laboratories, Burlingame, CA, USA) to counterstain nuclear DNA. Staining was performed on two wells per condition (i.e., IL-13-/+).

#### MUC5AC staining

After hydration in PBS, the cells were blocked for 10 min in 2.5% bovine serum albumin and 0.2% Triton X-100 diluted in PBS, followed by PBS wash and 1-h incubation at room temperature in primary antibody diluted in 0.25% bovine serum albumin and 0.05% Tween 20. Then, the cells were rinsed three times in PBS and incubated for 1 h at room temperature with the secondary antibody goat anti-rabbit Alexa Fluor 488 (1:300, A11034, Life Technologies) diluted in 0.25% bovine serum albumin and 0.05% Tween 20. After rinsing three times in PBS, the cells were mounted with VectaShield-DAPI (Vector Laboratories) to counterstain nuclear DNA.

Immunofluorescence detection of MUC5AC on bulbar conjunctival imprints was performed as described previously [34]. Briefly, primary antibody against MUC5AC and the secondary antibody Alexa Fluor 488 (1:300, A11034, Life Technologies) were diluted in 0.1% bovine serum albumin; after staining, the cells were mounted with VectaShield-DAPI (Vector Laboratories).

Immunofluorescent images were acquired with an Olympus BX51 microscope and CCD-1300 camera (VDS Vosskühler GmbH, Osnabrück, Germany). The images were analyzed with NIS Elements software (Laboratory Imaging). The percentage of positive cells was counted on six randomly captured photographs per well (two wells per condition) from at least four independent tissue donors.

### Quantitative real-time polymerase chain reaction (qPCR)

The expression of the *GAPDH* (glyceraldehyde-3-phosphate dehydrogenase), *TP63*, *MUC5AC*, *MUC4* (a conjunctiva-prevalent transmembrane mucin [5, 39]), *K3* and *K12* (keratins of differentiated corneal epithelial cells [40]), and *K7* genes were detected by qPCR. Cells from P0, P1 and P2 cultures were collected and transferred into Eppendorf tubes containing 500 μl TRI Reagent (Molecular Research Center, Cincinnati, OH, USA), and total RNA was extracted according to the manufacturer’s instructions. Total RNA (1 μg) was treated using deoxyribonuclease I (Promega, Madison, WI, USA) and used for reverse transcription (RT). First-strand complementary DNA (cDNA) was synthetized using M-MLV (Moloney murine leukemia virus) reverse transcriptase and random primers (Promega) in a total reaction volume of 25 μl. The qPCR was performed in a LightCycler 480 Real-Time PCR system (Roche, Basel, Switzerland).

Table 2 lists the primer sequences used for the amplification. The sequence specificity of the primers was confirmed via Primer-BLAST (National Center for Biotechnology Information). Conventional RT-PCR was performed to confirm that only a single band was obtained. The LightCycler 480 SYBR Green I Master (Roche) was used to perform the qPCR. The qPCR parameters involved denaturation at 95°C for 3 min, then 40 cycles at 95°C for 20 s, annealing at 60°C for 30 s, and elongation at 72°C for 15 s. Fluorescence was monitored at 55–95°C at 0.5°C intervals for 10 s. Triplicate reactions were performed for each sample and gene. A relative quantification model was used to calculate the expression of the target gene in comparison to the endogenous control *GAPDH*.

**Table 2:** Primers used for quantitative real-time PCR.

Gene (human)	Sequence (5′→3′)	GenBank accession number	Product size (bp)
*GAPDH*	F: GAAGGGGTCATTGATGGCAAC R: GGGAAGGTGAAGGTCGGAGTC	NM_001289746.1	108
*MUC5AC*	F: CCTGCAAGCCTCCAGGTAG R: CTGCTCCACTGGCTTTGG	NM_001304359.1	103
*MUC4*	F: TCCGTGTCCTGCTGGATAACC R: GTTGCGGCTCAGGAGGACTC	NM_018406.6	104
*TP63 (p63α* isomers)	F: GAGGTTGGGCTGTTCATCAT R: GAGGAGAATTCGTGGAGCTG	NM_001114980.1	174
*K3*	F: GGATGTGGACAGTGCCTATATG R: AGATAGCTCAGCGTCGTAGAG	NM_057088.2	106
*K7*	F: AGGATGTGGTGGAGGACTTC R: CTTGCTCATGTAGGCAGCAT	NM_005556.3	116
*K12*	F: CCAGGTGAGGTCAGCGTAGAA R: CCTCCAGGTTGCTGATGAGC	NM_000223.3	352

### Statistical analysis

Statistical analysis was carried out with GraphPad Prism (Graphpad Software, La Jolla, CA, USA). Descriptive statistics are reported as N (number of values), the mean ± SD, or the median with quartile range. Data distribution normality was assessed using the D’Agostino and Pearson omnibus normality test. Data sets that passed the normality test were analyzed using a two-tailed unpaired *t*-test (comparison between IL-13- and IL-13+ groups within passages) or one-way analysis of variance that in case of significance was followed by Bonferroni’s multiple comparison test (comparison among either IL-13- or IL-13+ groups). Data sets with lower numbers of values were analyzed using the two-tailed Mann-Whitney test (comparison between IL-13- and IL-13+ groups within passages) or Kruskal-Wallis test that in case of significance was followed by Dunn’s multiple comparison test (comparison among either IL-13- or IL-13+ groups). P-values < 0.05 were considered statistically significant.

## Results

**Fig 1:**
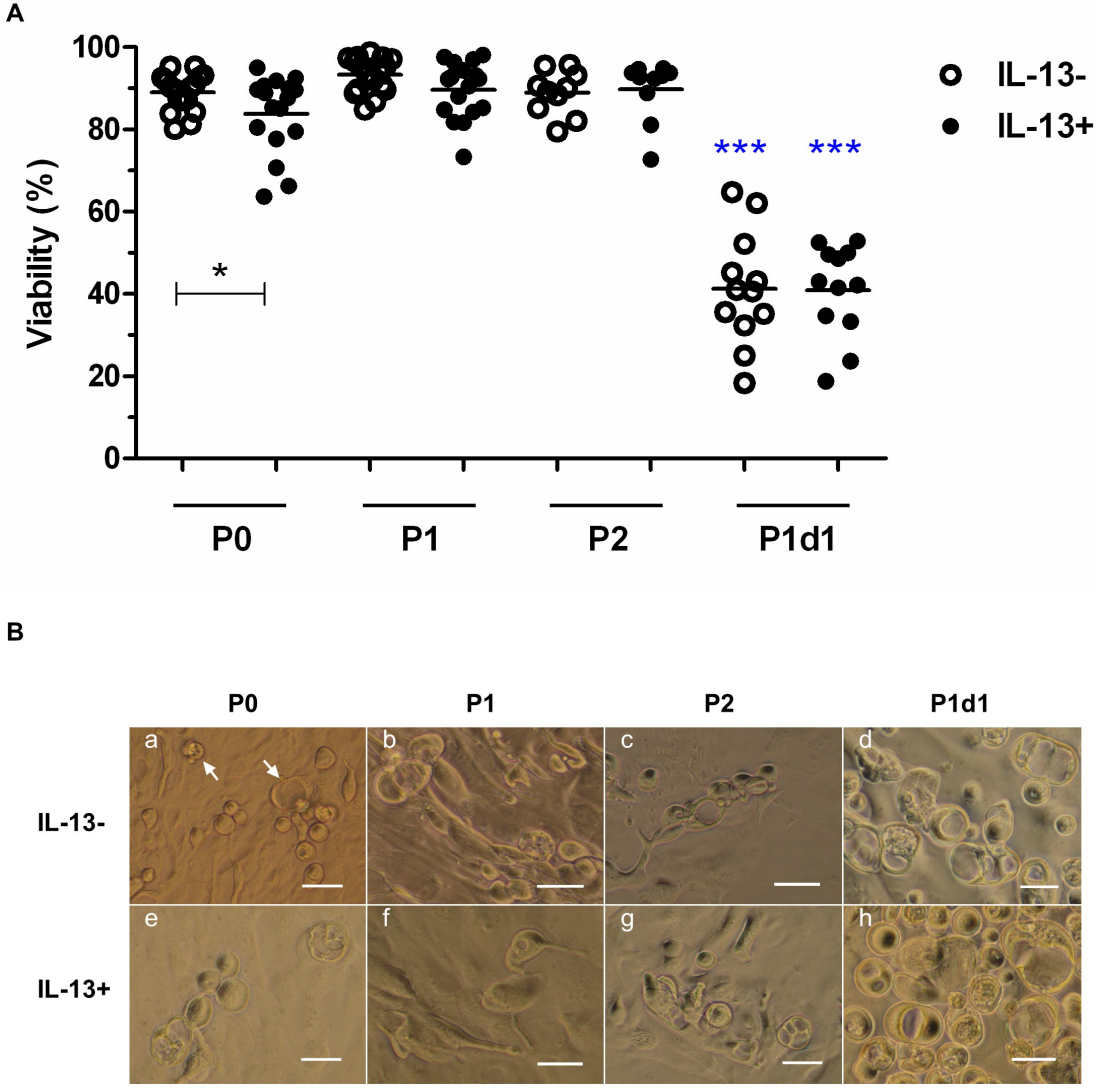
The viability and morphology of IL-13- or IL-13+ cell cultures. (A) Percentages of cell viability at the end of cultivation of P0, P1, and P2 cultures and percentages of cell viability of the unattached GC-enriched subpopulation harvested on P1d1. Cell viability data are presented in a vertical scatter plot graph with line indicating mean. *P < 0.05, ***P ≤ 0.001. Blue asterisks indicate significant decrease of viability in P1d1 IL-13- or P1d1 IL-13+ group vs. P0, P1, and P2 IL-13- or IL-13+ groups, respectively. (B) Cell morphology at the end of cultivation of P0, P1, and P2 cultures observed under inverted phase contrast microscope. P1d1 images show no attached GC-enriched subpopulations. White arrows show examples of GCs. Scale bars: 50 μm.

Cell outgrowth from limbal explants mostly began day 4 after culture initiation, and cells were 90–100% confluent at day 9, with no significant differences between the IL-13- and IL-13+ cultures. At the end of the P0 culture, the IL-13- group had significantly higher cell viability (89.06 ± 4.6%, N = 17, P = 0.0468) than the IL-13+ group (83.81 ± 9.4%, N = 17). At P1, IL-13- and IL-13+ cells reached confluence similarly and were harvested on day 9–12. The cell viability at the end of the P1 cultures was 91% (N = 17), with no statistical significance between groups. On P1d1, numerous unattached cells, particularly GCs, were seen in the IL-13- and IL-13+ cultures and were rinsed and evaluated. Their viability in both the IL-13- or IL-13+ groups was significantly lower (about 41%, N = 12, P ≤ 0.001) in comparison to viability of P0–P2 cultures. At P2, cells were harvested on day 12–14 of culture without reaching confluence. The cell viability was about 89% (N = 10), with no statistical significance between the IL-13- and IL-13+ groups (Fig 1A).

In P0, the epithelial cell morphology in the IL-13- and IL-13+ groups was cobblestone in shape and with a high nuclear-to-cytoplasmic ratio. Round to oval-shaped GCs were present either as single cells or as groups of cells. Most GCs were interspersed among epithelial cells and protruded above them (Fig 1Ba and 1Be). A mixture of cuboidal and flattened epithelial cells with superficially located GCs appeared at P1 (Fig 1Bb and 1Bf); differentiated flattened epithelial cells with low nuclear-to-cytoplasmic ratio and with superficially located GCs appeared at P2 (Fig 1Bc and 1Bg). On P1d1, many unattached cells were seen in the IL-13- and IL-13+ groups (Fig 1Bd and 1Bh).

### Characterization of cultured cells: Keratins

**Fig 2:**
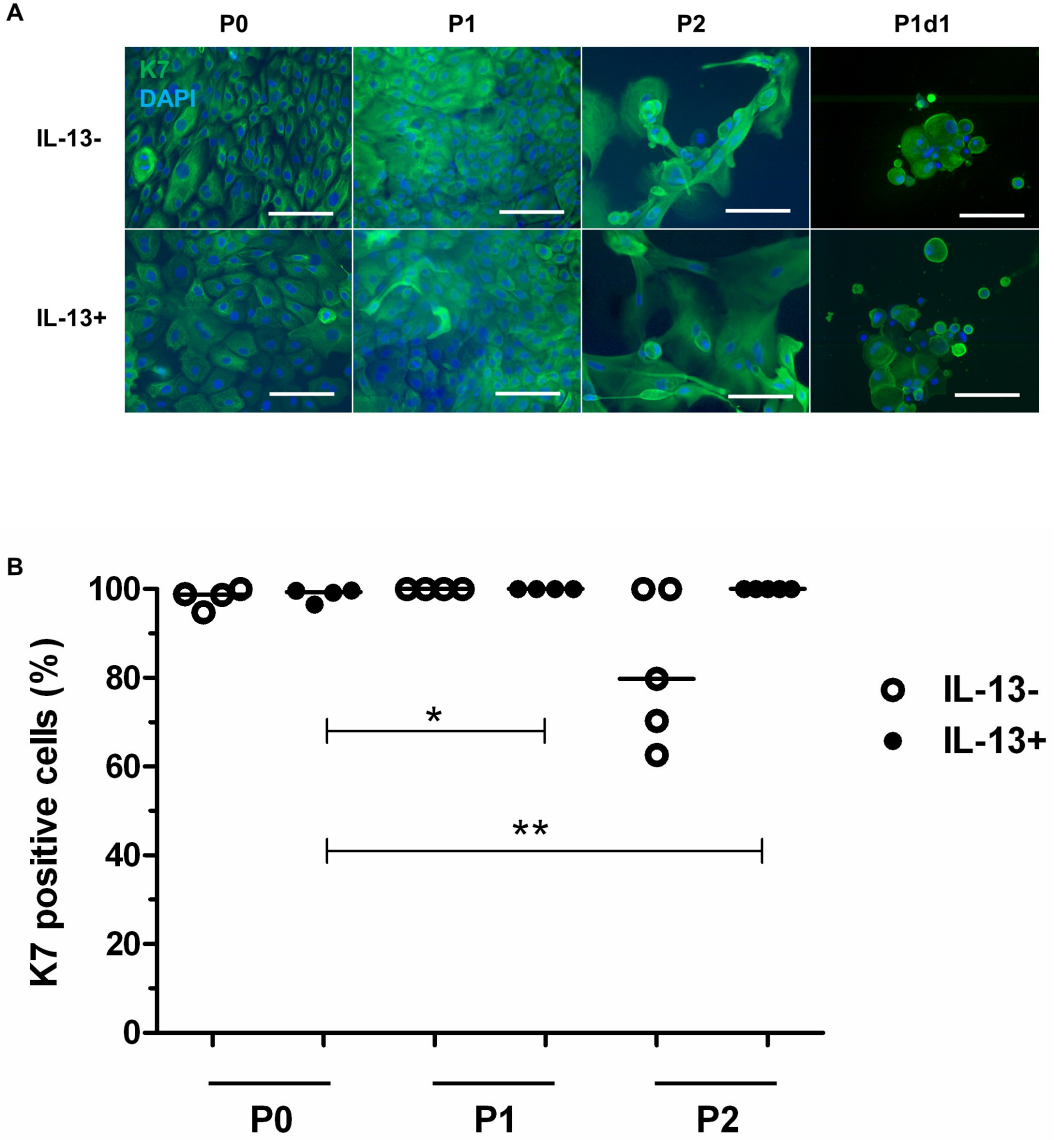
Immunofluorescent staining of K7 in IL-13- or IL-13+ cell cultures. P0 cells originating from limbal explants, P1 and P2 cells (all fixed at the end of cultivation), and the unattached GC-enriched subpopulation (P1d1, harvested on day 1 after passage of primary cells) were analyzed by immunofluorescent staining. (A) K7, green; DAPI-counterstained nuclei, blue; scale bars: 100 μm. (B) Distribution of percentages in P0, P1, and P2 groups for K7 positivity. All data of positive percentages are presented in the vertical scatter plot graph with line indicating median. *P < 0.05, **P ≤ 0.01.

Strong K7 immunostaining clearly showed conjunctival epithelial cells and GCs in IL-13- and IL-13+ cultures at the end of the P0, P1, and P2 cultivation periods and in the P1d1 GC-enriched population (Fig 2A). There were >99% K7-positive cells in the P0 and P1 IL-13- and IL-13+ groups and in the P2 IL-13+ group; K7 positivity was 80% only in the P2 IL-13- group (Fig 2B, descriptive statistics in S1 Table). K7 positivity increased significantly in the P1 IL-13+ (P < 0.05) and P2 IL-13+ (P ≤ 0.01) groups in comparison to the P0 IL-13+ group.

**Fig 3:**
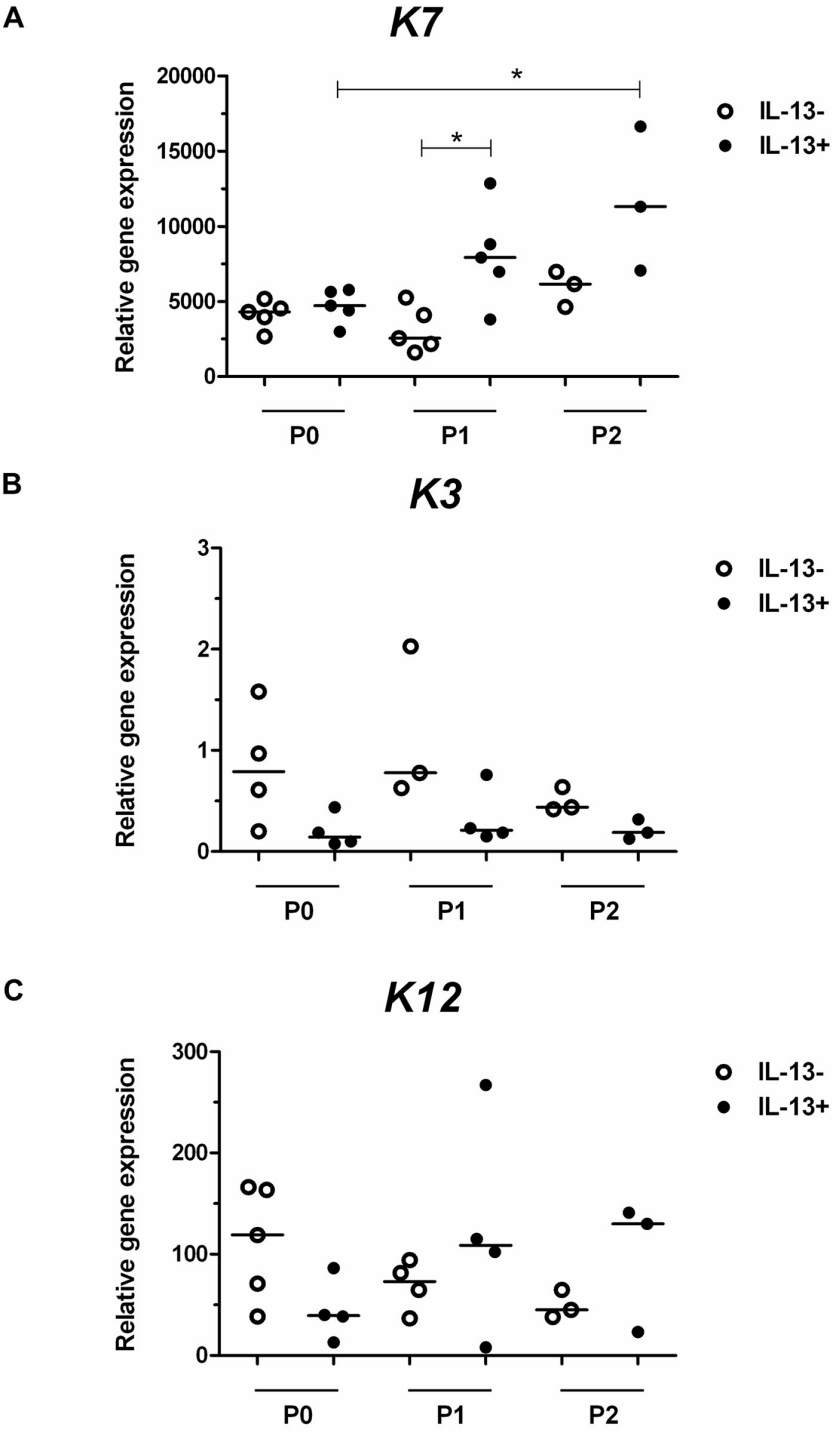
Relative expression of *K7*, *K3*, and *K12* genes in IL-13- or IL-13+ cell cultures. At the end of cultivation, P0 cells originating from limbal explants and passaged cells (P1 and P2) were analyzed for *K7* (A), *K3* (B), and *K12* (C) gene expression by qPCR. All data are presented in a vertical scatter plot graph with line indicating median. *P < 0.05.

Gene expression was measured for the *K7*, *K3*, and *K12* genes. Strong *K7* expression was present in IL-13- and IL-13+ conditions and in all evaluated groups (Fig 3A). The median values of *K7* expression were higher in all passages of IL-13+ cells, and that in the P1 IL-13+ group was significantly different (P = 0.0317) compared to the P1 IL-13- group. In the IL-13+ cultures, *K7* expression was significantly higher (P < 0.05) at the end of P2 compared to P0. *K3* and *K12* genes were expressed in all tested groups (Fig 3B and 3C, respectively) but at much lower levels, especially low expression was seen for *K3*. S2 Table presents the descriptive statistics of *K7*, *K3*, and *K12* gene expression in the P0, P1, and P2 groups.

### Characterization of cultured cells: Mucins

**Fig 4:**
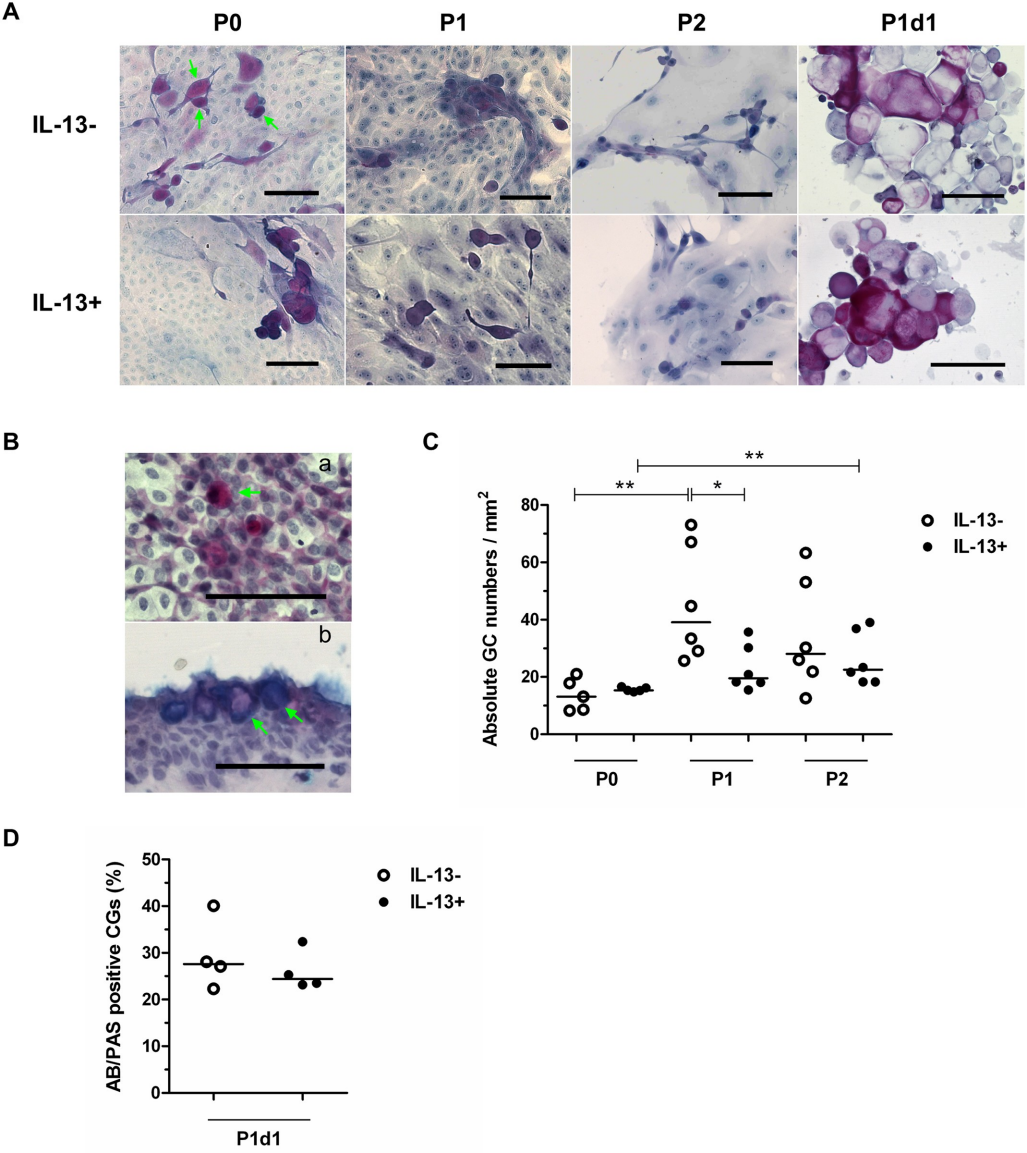
AB/PAS staining of GCs in IL-13- or IL-13+ cell cultures and P1d1 subpopulation. (A) P0 cells originating from limbal explants, P1 and P2 cells (all fixed at the end of cultivation), and the unattached GC-enriched subpopulation (P1d1, harvested on day 1 after passage of primary cells) were analyzed by AB/PAS staining. Green arrows indicate examples of GCs. (B) AB/PAS-positive GCs on the surface of the conjunctiva (impression cytology) (a) and on pterygium cryosections (b) were used as a positive control. Green arrows indicate examples of GCs. (C) Distribution of absolute numbers of AB/PAS-positive GCs in individual groups presented in a vertical scatter plot graph with line indicating median. (D) Distribution of percentages of AB/PAS-positive GCs in individual groups of P1d1 subpopulation presented in a vertical scatter plot graph with line indicating median. *P < 0.05, **P ≤ 0.01. Scale bars: 100 μm.

AB/PAS staining revealed the presence of single and grouped GCs in all groups under IL-13- or IL-13+ conditions (Fig 4A). Pure neutral mucins and a mixture of neutral and acidic mucins were produced in the P0, P1, and P2 cultures and within the P1d1 GC-enriched subpopulation. The presence of only acidic mucins in GCs was very rare. Bulbar conjunctival impression cytology specimens and cryosectioned pterygium specimens served as a positive control for AB/PAS staining (Fig 4B). AB/PAS-positive GCs were counted and are expressed as medians of absolute cell numbers/mm^2^. The absolute number of GCs in the P1 IL-13- group was significantly higher (P = 0.0411) than that in the P1 IL-13+ group. Among the IL-13- groups, significantly higher numbers of GCs were present at P1 (P ≤ 0.01) in comparison to P0; among the IL-13+ groups, there were significantly higher numbers of GCs at P2 (P ≤ 0.01) in comparison to P0 (Fig 4C). S3 Table shows the descriptive statistics of the AB/PAS-positive GCs.

P1d1 GC-enriched population showed AB/PAS-positivity in 27.6% of cells in IL-13- group and 24.4% of cells in IL-13+ group with no significant difference between groups (Fig 4D). S4 Table shows the descriptive statistics of the AB/PAS-positive GCs in P1d1 population.

**Fig 5:**
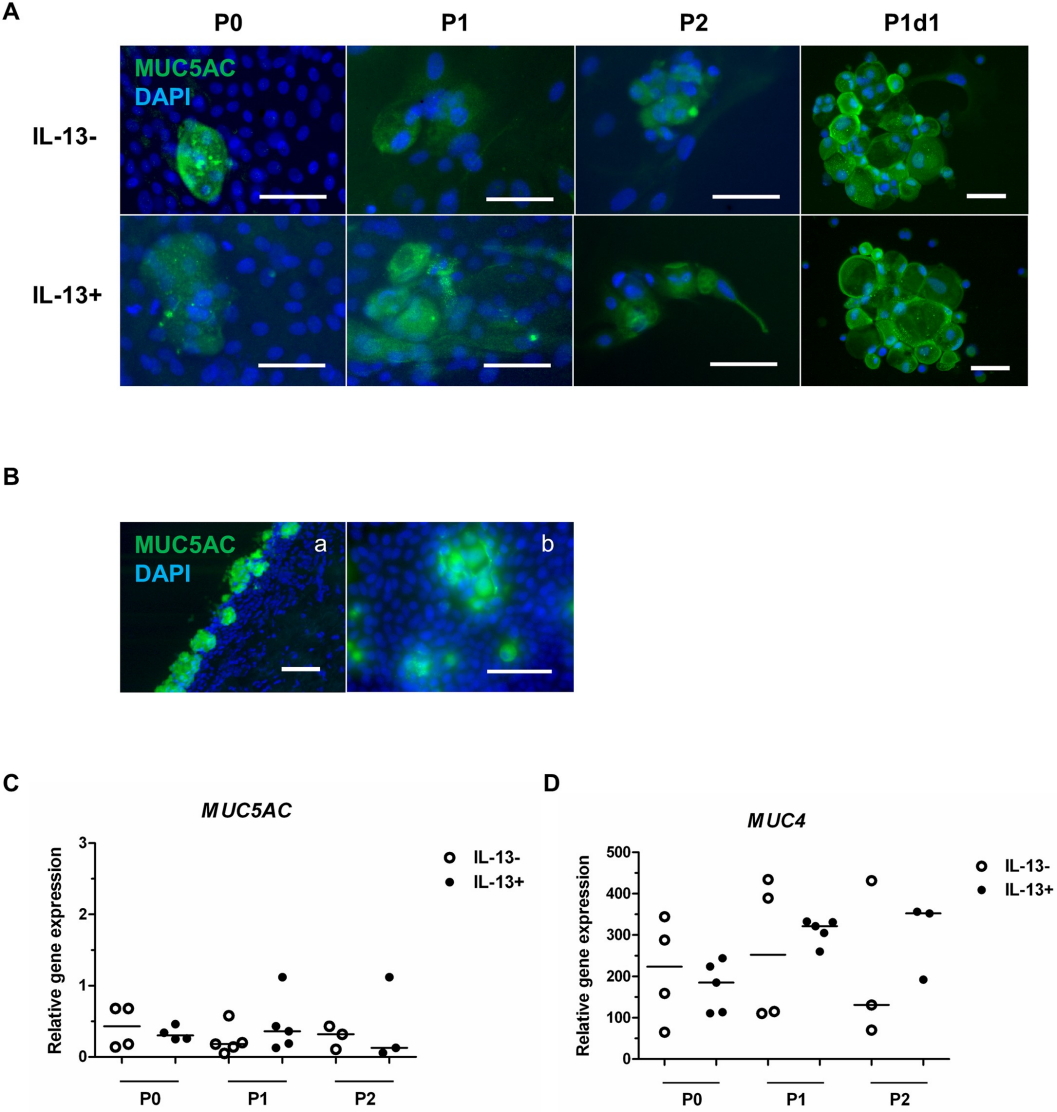
Immunofluorescent staining of MUC5AC and relative expression of *MUC5AC* and *MUC4* genes in IL-13- or IL-13+ cell cultures. (A) P0 cells originating from limbal explants, P1 and P2 cells (all fixed at the end of cultivation), and the unattached GC-enriched subpopulation (P1d1, harvested on day 1 after passage of primary cells) were analyzed by immunofluorescent staining for MUC5AC (green). Nuclei were counterstained with DAPI (blue). (B) MUC5AC staining of GCs on pterygium cryosection (a) and upper bulbar conjunctival impression cytology (b). qPCR analysis of the relative gene expression of (C) *MUC5AC* and (D) *MUC4*. All data are presented in vertical scatter plot graphs with line indicating median. Scale bars: 50 μm.

MUC5AC immunostaining confirmed the presence of single and grouped GCs in all tested groups (Fig 5A). Cells harvested by bulbar impression cytology and pterygium sections were used as positive controls for MUC5AC staining (Fig 5B). qPCR confirmed *MUC5AC* gene expression, which was present in all evaluated groups but was not statistically significant among groups (Fig 5C, S2 Table). *MUC4* was expressed in all groups under IL-13- and IL-13+ conditions but with no statistical significance among groups (Fig 5D, S2 Table).

### Characterization of cultured cells: Proliferation and stemness

**Fig 6:**
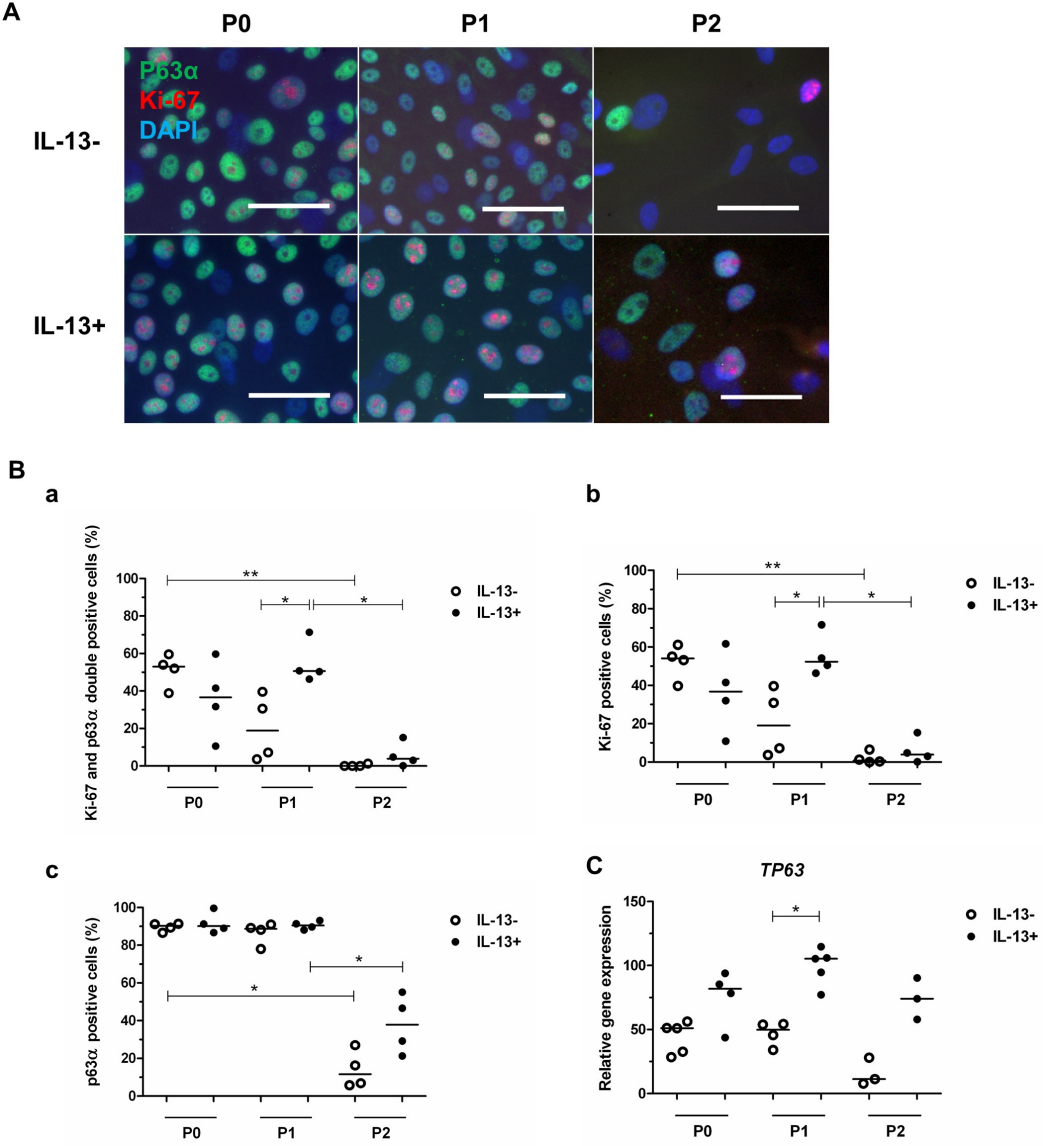
Immunofluorescent Ki-67 and p63α double staining and the relative *TP63* gene expression in IL-13- or IL-13+ cell cultures. (A) At the end of cultivation, P0 cells originating from limbal explants and P1 and P2 cells were analyzed by immunofluorescent staining for Ki-67 (red) and p63α (green); nuclei were counterstained with DAPI (blue). Scale bars: 50 μm. (B) Distribution of percentages in P0, P1, and P2 groups for Ki-67 and p63α double staining (a), and Ki-67 (b) and p63α (c) immunostaining. (C) qPCR analysis of relative *TP63* gene expression. All data are presented in vertical scatter plot graphs with line indicating median. *P < 0.05, **P ≤ 0.01.

Ki-67 and p63α immunostaining demonstrated a high percentage of positivity, particularly in the P0 and P1 groups, and a low percentage of positivity in the P2 groups (Fig 6A). The P0 IL-13- (53%) and P1 IL-13+ (51%) groups had the highest Ki-67 and p63α double positivity, while expression was lowest (<4%) in the P2 IL-13- (P ≤ 0.01) and IL-13+ (P < 0.05) groups. Between the P0, P1, and P2 IL-13- and IL-13+ groups, P1 IL-13+ cells had significantly higher (P = 0.0286) Ki-67 and p63α double positivity (Fig 6Ba, S1 Table).

A similar pattern of antigen expression was seen for Ki-67 staining versus Ki-67 and p63α double staining; indicating almost 100% Ki-67 co-localization with p63α in P0, P1, and P2 cells (Fig 6Bb, S1 Table).

p63α immunostaining was present in around 90% of cells in the P0 and P1 groups, with a significant decrease (P < 0.05) in p63α positivity to 12% in the P2 IL-13- group and to 38% in the P2 IL-13+ group. No difference was seen between the P0, P1, and P2 IL-13- and IL-13+ groups (Fig 6Bc, S1 Table).

*TP63* gene expression was present in all evaluated groups under the IL-13- and IL-13+ conditions (Fig 6C, S2 Table). All IL-13+ groups had higher median *TP63* expression, with the P1 IL-13+ group having significantly higher median *TP63* expression (P = 0.0159) than the P1 IL-13- group.

### Colony-forming efficiency

**Fig 7:**
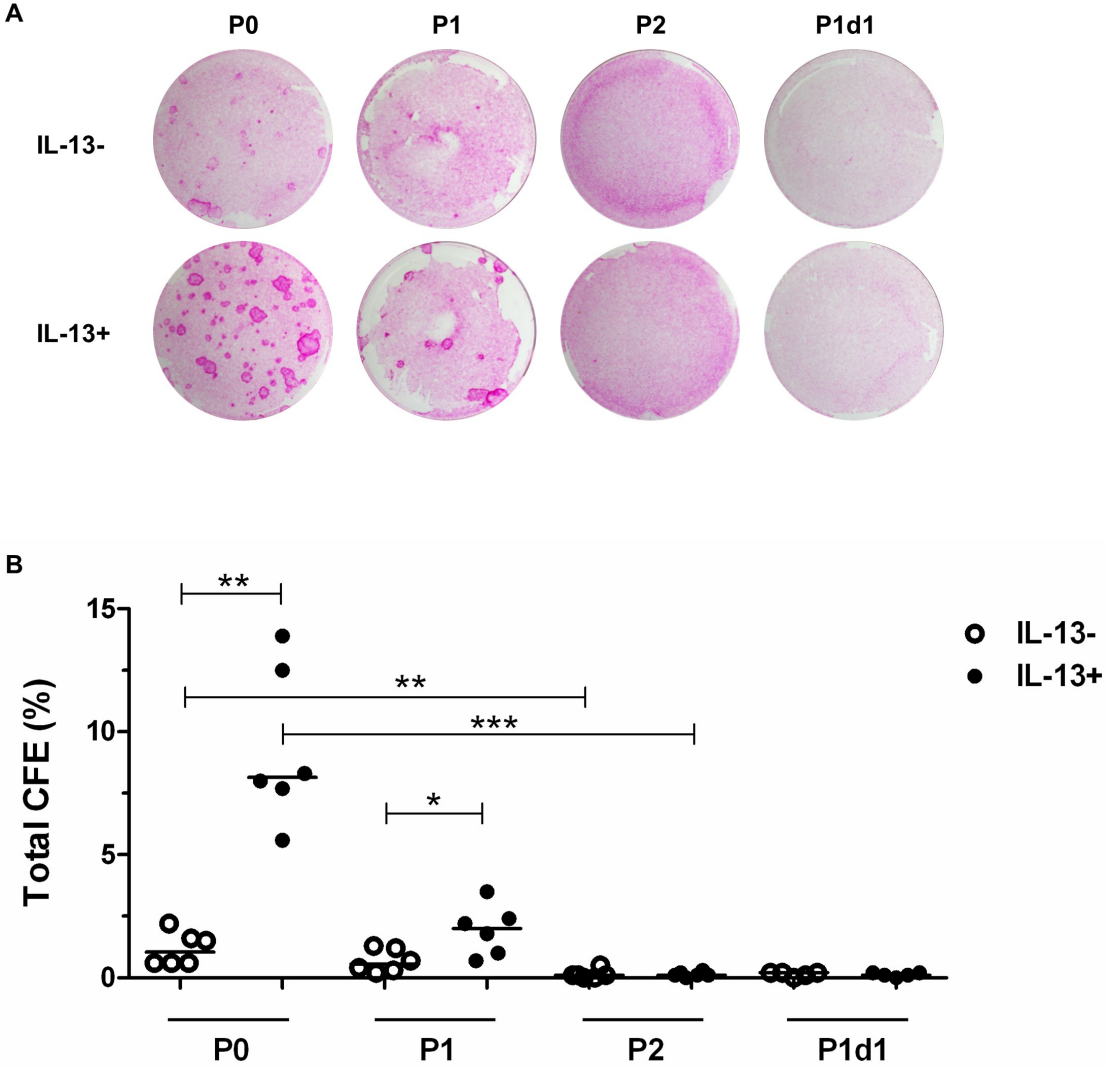
Comparison of total CFE. At the end of cultivation, P0 cells from limbal explants, P1 and P2 cells, and the unattached GC-enriched subpopulation (P1d1, harvested on day 1 after passage of primary cells) were cultured with growth-arrested 3T3 mouse fibroblasts to compare their growth ability under IL-13- and IL-13+ conditions. All total CFE data are presented in vertical scatter plot graphs with line indicating median. *P < 0.05, **P ≤ 0.01, ***P ≤ 0.001. (A) Colonies grown in CFE assay and stained with 2% rhodamine B (day 12). (B) Distribution of total CFE percentages of the P0, P1, P2, and P1d1 groups.

The P0 IL-13- group had about 1% total CFE; that of the P0 IL-13+, P1 IL-13-, and P1 IL-13+ groups were about 8%, 0.5%, and 2%, respectively; in the P2 and P1d1 IL-13- and IL-13+ groups, the total CFE was <0.5% (Fig 7). Statistical analysis of the CFE data demonstrated higher growth potential in P0 IL-13- (P ≤ 0.01) or IL-13+ cultures (P ≤ 0.001) compared to that of consequent passages, especially P2. The P0 IL-13+ group had significantly higher growth potential (P = 0.0048) compared to the P0 IL-13- group. Similarly, the P1 IL-13+ group had significantly higher growth potential (P = 0.037) than the P1 IL-13- group (Fig 7B, S5 Table).

## Discussion

We successfully prepared conjunctival epithelium using limbal explants from surplus tissue from human corneoscleral rims. The cultured epithelium was composed of K7-positive epithelial cells and GCs; additionally, the GCs showed AB/PAS and MUC5AC positivity and *MUC5AC* expression. Previously published studies have reported inconsistent results, as they have demonstrated either presence or absence of GCs in cultures derived from human limbal cells [7, 41]. Conjunctival cultures did not contain GCs if explants were obtained <3 mm from the limbus [42], but GCs were present in cultures if the biopsy was obtained 3–5 mm from the corneoscleral rim [43].

Although the number of GCs in our cultures appears low, about 13–15 GCs/mm^2^, they are markedly higher compared to the 0.5–0.6 GCs/mm^2^ reported by Ang *et al*., who used explants from the superior bulbar region [44], and lie between the GC levels obtained by Pellegrini *et al*. in non-confluent and confluent cultures, respectively [7]. Using flow cytometry, Lužnik *et al*. reported a relatively high percentage of GCs (9–11% based on serum concentration) [41] in limbal explant cultures, which could be an interesting result; unfortunately, their results are expressed as the mean ± standard error of the mean, which does not adequately describe the actual dispersion of values [45]. Moreover, their error bars are quite large despite the standard error of the mean, which presumes large variability in the distribution. Concerning the P1 cultures, the P1 IL-13- group had more GCs, i.e., 39 GCs/mm^2^, and the P1 IL-13+ group had similar GC numbers, i.e., 19 GCs/mm^2^, as compared to the P1 control group of human conjunctival epithelial cells of Schrader *et al*. [43].

The positive influence of IL-13 on GC numbers [19, 20] and MUC5AC secretion has previously been reported [22]. In the present study, we did not observe differences in the number of AB/PAS-positive GCs between the P0 IL-13- and IL-13+ cultures. From this point of view, IL-13 in our P0 cultures did not influence the number of GCs as compared to two studies in mice [19, 20], which achieved the proliferation of conjunctival GCs. However, the two studies describe cultures that are far from the natural GC-to-epithelial cell ratio [7], as the reported GC content was incredibly high, showing GC presence in cultures as high as 100% [19] and 85% [20]. Differences between animal and human studies are not surprising, as it has been proposed that human conjunctival GCs are post-mitotic terminally differentiated cells [7], while mouse conjunctival GCs have mitotic activity [8]. Surprisingly, not only did IL-13 not increase GC numbers in our cultures, there were significantly more AB/PAS-positive GCs in the P1 IL-13- group. Thus, it appears that IL-13 prevents the differentiation of young transient cells into GCs, and this finding might support the fact that IL-13 maintained stemness in our cultures. Here, we evaluated for the first time the relationship between IL-13 and the gene expression of the human conjunctival mucins *MUC5AC* and *MUC4*, and found that, at the end of the P0, P1, and P2 cultures, IL-13 did not alter their expression.

The determination of GC numbers was done using AB/PAS staining because it is easier to distinguish individual GCs within cell clusters with AB/PAS staining than with MUC5AC staining; additionally, histological staining yielded better information on the morphology of our cultures. On P1d1 under IL-13- and IL-13+ conditions, we observed cells with typical GC morphology, and these spontaneously unattached cells were collected and characterized by AB/PAS and MUC5AC staining. AB/PAS staining revealed about 28% and 24% of positive cells in IL-13- and IL-13+ group, respectively. This P1d1 GC-enriched subpopulation did not exhibit clonogenic ability, which is consistent with the proposal that human conjunctival GCs are terminally differentiated [7], especially if they produce MUC5AC [3, 46]. Moreover, the GC lifespan in culture appeared quite short, as we observed floating detached GCs daily. The lifespan of conjunctival GCs has not been studied so far; however, for example, intestinal GC turnover is 3–7 days [47].

The presence of the conjunctival cell marker K7 throughout the cultivation is consistent with the conjunctival, but not corneal phenotype of epithelial cells [3, 34]. Moreover, our results clearly show that K7 was present in both conjunctival cell types, i.e., in epithelial cells and GCs. In the present study, the IL-13+ cell cultures had significantly higher *K7* expression and more histologically stained GCs in P2 cells compared to P0 cells. This finding is consistent with the increasing differentiation observed throughout P0, P1, and P2.

As we cultured limbal explants that are primarily considered as a source for corneal tissue, we tested our cultures for the cornea-specific genes *K3* and *K12*, which confirmed their expression. We also showed that IL-13 does not alter their expression significantly. However, the difference between *K3* (very low) and *K12* (higher) expression was present. Although K3 and K12 form a pair at a protein level, at the mRNA level, they are encoded by different genes and located at different chromosomes. Moreover, their expression is induced by two different PAX6 isoforms and enhanced by different factors [48]. Therefore, the difference between the expressions of these two genes is possible, and lower relative gene expression of *K3* versus *K12* in corneal, limbal and conjunctival tissue was shown [48]. Human conjunctival epithelium contains ectopically residing clusters of K12-positive epithelial cells [49], conjunctival cultures initiated from cells from the proximity of the limbal area express *K3* and *K12* [42], and corneal and conjunctival lineages both come from PAX6 ectodermal origin [50]. Thus, the mixed expression of corneal and conjunctival markers in our limbal tissue–derived cultures is not surprising. However, due to the predominant expression of *K7* over *K12* and *K3*, we believe that our cultures differentiated primarily to the conjunctival phenotype, which is also supported by the expression of conjunctiva-prevalent *MUC4* and GCs presence. Previously, we found that K7-positivity in human limbal explant cultures also appeared but it was lower to K3- and K12-positivity [51]. Tissue with conjunctival markers cultured from human limbal explants has been also prepared by Luznik *et al*. [41]. They found that higher percentage of used human serum lead to higher expression of K7 and MUC5AC in limbal explant cultures. Thus, the appearance of conjunctival markers in our cultures could also be associated with usage of serum (FBS). For example, one of active serum components, nerve growth factor (NGF) increases the presence of GCs and MUC5AC expression in mouse limbal cultures [10] and increases goblet cell number and their differentiation in human conjunctival cultures [52]. However, the concentration of NGF in serum is much lower (pg/ml) compared to efficient NGF concentration used *in vitro* (ng/ml) [52, 53].

Currently, p63α is considered the most important marker characterizing limbal stem cells and young transient amplifying cells that give rise to holoclones and meroclones, respectively [37, 38]. Clinical results have shown that limbal transplants containing >3% p63-bright cells led to successful corneal epithelial regeneration in a higher percentage of eyes with limbal stem cell deficiency (78%) than transplants with ≤3% p63-bright cells (11%) [32]. In the present study, the P0 and P1 cultures contained very high percentages of p63α-positive cells (about 90%), although the IL-13- and IL-13+ groups were not significantly different; however, the IL-13+ groups had increased *TP63* expression, especially the P1 groups, and demonstrating that IL-13 maintained the stemness of the cultures. p63α is expressed in cells with high proliferative potential that are slow-cycling *in vivo* but extensively proliferating *in vitro* [38]. p63α and Ki-67 double staining showed the number of p63α-positive cells that had proliferated at the end of the culture period, and double staining positivity was highest in the P0 IL-13- (53%) and P1 IL-13+ groups (51%). The P1 IL-13- group had a higher percentage of Ki-67–positive proliferating cells (19%) than conjunctival cultures seeded in plasma or cryoprecipitate scaffolds (P1, ~11%) [22] and a lower percentage than that in P1 cells in another study (39%) [54]. The increased Ki-67 positivity (52%) in the P1 IL-13+ groups demonstrates the effect of IL-13 on epithelial cell proliferation.

Our P0 IL-13- cultures had comparable clonogenic ability (1%) with those of some areas of human conjunctival tissue [9, 54] and limbal explants [55]. IL-13 can inhibit or stimulate colony formation depending on cell type and dose [56, 57]. In the P0 IL-13+ cultures, clone-forming ability increased to 8%, which was even higher than that of cultures from the human inferior forniceal and medial canthal areas [9]. The higher clonogenic capacity of IL-13–stimulated epithelial cells in P0 and P1 corresponds with the same tendency shown in *TP63* expression, particularly in P1. Of note, IL-13 also preserved K7 expression in our cultures. Thus, it appears IL-13 has a two-fold effect; the maintenance of stemness and the support of differentiation. Differentiation of limbal epithelial cells requires asymmetric cell division [58]. Therefore, the more stem cells present in the cell culture, the more differentiated cells will be generated (IL-13+ cultures). Conversely, if the number of stem cells decreases during cultivation, the number of differentiated cells will also decrease as terminally differentiated cells do not have the proliferation activity (IL-13- cultures).

In conclusion, we have cultured human limbal explants prepared from corneoscleral rims and engineered epithelium with a predominant conjunctival phenotype with the presence of stem/progenitor/proliferating cells and a relatively high density of GCs. We show that IL-13 maintains the stemness of the cultures by increasing their clonal ability and *TP63* expression. IL-13 also preserved the expression of conjunctiva-specific keratin (K7) during passage, with no other significant changes in conjunctival mucin expression, GC number, and cornea-specific keratins. Moreover, we have isolated a subpopulation containing GCs and have demonstrated that mucin-producing GCs are terminally differentiated cells with no proliferative potential. For the first time, we raise the possibility of using corneoscleral rims as an alternative source for engineering a conjunctival epithelium that can be used for further research on GCs and for treating patients with ocular surface disorders.

## Supporting information

S1 Table Descriptive statistics of indirect fluorescent immunocytochemistry. (DOCX)

S2 Table Descriptive statistics of relative gene expression. (DOCX)

S3 Table Descriptive statistics of absolute GC numbers per mm^2^. (DOCX)

S4 Table Descriptive statistics of AB/PAS-positive GCs in P1d1 subpopulation. (DOCX)

S5 Table Descriptive statistics of total CFE. (DOCX)
