# Supplementary material for: Interleukin-13 maintains the stemness of conjunctival epithelial cell cultures prepared from human limbal explants
Source: PLoS One. 2019 Feb 11;14(2):e0211861. doi: 10.1371/journal.pone.0211861 (PMC6370187; doi:10.1371/journal.pone.0211861)
Supplement: S1 Table — (DOCX) [file pone.0211861.s001.docx]

| **Table S1** Descriptive statistics of indirect fluorescent immunocytochemistry | | | | | | | |
| --- | --- | --- | --- | --- | --- | --- | --- |
|  |  | **P0 IL-13-** | **P0 IL-13+** | **P1 IL-13-** | **P1 IL-13+** | **P2 IL-13-** | **P2 IL-13+** |
| **K7 (%)** | Number of values | 4 | 4 | 4 | 4 | 5 | 5 |
|  | Minimum | 94.80 | 96.50 | 100.00 | 100.00 | 62.60 | 100.00 |
|  | 25% Percentile | 95.78 | 97.18 | 100.00 | 100.00 | 66.45 | 100.00 |
|  | **Median** | **98.80** | **99.40** | **100.00** | **100.00** | **79.80** | **100.00** |
|  | 75% Percentile | 99.73 | 99.68 | 100.00 | 100.00 | 100.00 | 100.00 |
|  | Maximum | 100.00 | 99.70 | 100.00 | 100.00 | 100.00 | 100.00 |
| **Ki-67 (%)** | Number of values | 4 | 4 | 4 | 4 | 4 | 4 |
|  | Minimum | 39.70 | 10.90 | 3.70 | 46.40 | 0.00 | 0.10 |
|  | 25% Percentile | 43.08 | 16.18 | 4.58 | 47.43 | 0.08 | 0.83 |
|  | **Median** | **54.10** | **36.75** | **19.05** | **52.35** | **0.80** | **3.90** |
|  | 75% Percentile | 59.58 | 56.65 | 37.43 | 67.25 | 5.20 | 12.75 |
|  | Maximum | 61.10 | 61.70 | 39.60 | 71.60 | 6.50 | 15.40 |
| **p63α (%)** | Number of values | 4 | 4 | 4 | 4 | 4 | 4 |
|  | Minimum | 86.60 | 86.70 | 78.00 | 88.10 | 5.80 | 21.30 |
|  | 25% Percentile | 87.30 | 87.27 | 80.55 | 88.50 | 6.08 | 23.28 |
|  | **Median** | **90.30** | **90.15** | **88.70** | **90.55** | **11.60** | **37.90** |
|  | 75% Percentile | 91.35 | 97.53 | 90.55 | 92.68 | 24.33 | 52.98 |
|  | Maximum | 91.40 | 99.60 | 91.00 | 93.10 | 27.00 | 55.10 |
| **Ki-67p63α (%)** | Number of values | 4 | 4 | 4 | 4 | 4 | 4 |
|  | Minimum | 38.90 | 10.60 | 3.60 | 46.40 | 0.00 | 0.10 |
|  | 25% Percentile | 42.18 | 15.88 | 4.50 | 47.40 | 0.00 | 0.83 |
|  | **Median** | **53.00** | **36.60** | **18.90** | **50.60** | **0.00** | **3.90** |
|  | 75% Percentile | 58.20 | 55.15 | 37.35 | 66.25 | 0.98 | 12.60 |
|  | Maximum | 59.60 | 59.70 | 39.60 | 71.40 | 1.30 | 15.20 |
