# Supplementary material for: Interleukin-13 maintains the stemness of conjunctival epithelial cell cultures prepared from human limbal explants
Source: PLoS One. 2019 Feb 11;14(2):e0211861. doi: 10.1371/journal.pone.0211861 (PMC6370187; doi:10.1371/journal.pone.0211861)
Supplement: S2 Table — (DOCX) [file pone.0211861.s002.docx]

| **TABLE S2**: Descriptive statistics of relative gene expression | | | | | | | |
| --- | --- | --- | --- | --- | --- | --- | --- |
|  |  | **P0 IL-13-** | **P0 IL-13+** | **P1 IL-13-** | **P1 IL-13+** | **P2 IL-13-** | **P2 IL-13+** |
| ***K7* (%)** | Number of values | 5 | 5 | 5 | 5 | 3 | 3 |
|  | Minimum | 2667.00 | 2987.00 | 1614.00 | 3816.00 | 4649.00 | 7055.00 |
|  | 25% Percentile | 3309.00 | 3700.00 | 1895.00 | 5395.00 | 4649.00 | 7055.00 |
|  | **Median** | **4303.00** | **4730.00** | **2553.00** | **7928.00** | **6156.00** | **11316.00** |
|  | 75% Percentile | 4862.00 | 5704.00 | 4680.00 | 10835.00 | 6974.00 | 16644.00 |
|  | Maximum | 5176.00 | 5770.00 | 5261.00 | 12864.00 | 6974.00 | 16644.00 |
| ***K3* (%)** | Number of values | 4 | 4 | 3 | 4 | 3 | 3 |
|  | Minimum | 0.20 | 0.08 | 0.63 | 0.15 | 0.42 | 0.13 |
|  | 25% Percentile | 0.30 | 0.09 | 0.63 | 0.16 | 0.42 | 0.13 |
|  | **Median** | **0.79** | **0.15** | **0.78** | **0.21** | **0.44** | **0.19** |
|  | 75% Percentile | 1.43 | 0.38 | 2.03 | 0.63 | 0.64 | 0.32 |
|  | Maximum | 1.58 | 0.44 | 2.03 | 0.76 | 0.64 | 0.32 |
| ***K12* (%)** | Number of values | 5 | 4 | 4 | 4 | 3 | 3 |
|  | Minimum | 38.52 | 13.13 | 36.96 | 8.04 | 38.04 | 23.39 |
|  | 25% Percentile | 54.88 | 19.50 | 43.92 | 31.62 | 38.04 | 23.39 |
|  | **Median** | **119.20** | **39.39** | **73.21** | **108.80** | **45.08** | **130.20** |
|  | 75% Percentile | 165.00 | 74.76 | 91.14 | 229.10 | 64.94 | 141.20 |
|  | Maximum | 166.30 | 86.29 | 94.31 | 267.10 | 64.94 | 141.20 |
| ***MUC5AC* (%)** | Number of values | 4 | 4 | 5 | 5 | 3 | 3 |
|  | Minimum | 0.14 | 0.25 | 0.05 | 0.13 | 0.11 | 0.06 |
|  | 25% Percentile | 0.15 | 0.25 | 0.10 | 0.16 | 0.11 | 0.06 |
|  | **Median** | **0.43** | **0.30** | **0.18** | **0.36** | **0.32** | **0.13** |
|  | 75% Percentile | 0.68 | 0.43 | 0.39 | 0.78 | 0.43 | 1.12 |
|  | Maximum | 0.68 | 0.46 | 0.58 | 1.12 | 0.43 | 1.12 |
| ***MUC4*  (%)** | Number of values | 4 | 5 | 4 | 5 | 3 | 3 |
|  | Minimum | 65.39 | 111.00 | 110.50 | 260.40 | 70.41 | 192.40 |
|  | 25% Percentile | 88.74 | 112.30 | 111.70 | 282.90 | 70.41 | 192.40 |
|  | Median | **223.50** | **185.40** | **252.20** | **321.30** | **131.10** | **352.40** |
|  | 75% Percentile | 330.30 | 234.10 | 423.10 | 331.90 | 431.40 | 356.50 |
|  | Maximum | 344.40 | 244.10 | 434.40 | 332.60 | 431.40 | 356.50 |
| ***TP63* (%)** | Number of values | 5 | 4 | 4 | 5 | 3 | 3 |
|  | Minimum | 28.40 | 43.74 | 34.08 | 77.23 | 7.82 | 57.99 |
|  | 25% Percentile | 30.55 | 52.38 | 36.99 | 85.94 | 7.82 | 57.99 |
|  | **Median** | **51.07** | **81.80** | **49.82** | **105.30** | **11.27** | **74.08** |
|  | 75% Percentile | 53.73 | 91.82 | 54.25 | 110.30 | 28.04 | 90.37 |
|  | Maximum | 56.27 | 93.99 | 54.36 | 114.70 | 28.04 | 90.37 |
