# Supplementary material for: Interleukin-13 maintains the stemness of conjunctival epithelial cell cultures prepared from human limbal explants
Source: PLoS One. 2019 Feb 11;14(2):e0211861. doi: 10.1371/journal.pone.0211861 (PMC6370187; doi:10.1371/journal.pone.0211861)
Supplement: S3 Table — (DOCX) [file pone.0211861.s003.docx]

| **TABLE S3** Descriptive statistics of absolute GC numbers per mm^2^ | | | | | | | |
| --- | --- | --- | --- | --- | --- | --- | --- |
|  |  | **P0 IL-13-** | **P0 IL-13+** | **P1 IL-13-** | **P1 IL-13+** | **P2 IL-13-** | **P2 IL-13+** |
| **AB/PAS** | Number of values | 5 | 5 | 6 | 6 | 6 | 6 |
|  | Minimum | 8.30 | 14.80 | 25.70 | 15.50 | 12.60 | 18.30 |
|  | 25% Percentile | 8.45 | 15.05 | 28.25 | 17.45 | 19.58 | 18.30 |
|  | **Median** | **13.20** | **15.40** | **39.10** | **19.60** | **28.10** | **22.55** |
|  | 75% Percentile | 19.45 | 16.40 | 68.60 | 31.58 | 55.65 | 37.43 |
|  | Maximum | 21.00 | 16.60 | 73.10 | 35.70 | 63.30 | 39.00 |
