# Supplementary material for: Interleukin-13 maintains the stemness of conjunctival epithelial cell cultures prepared from human limbal explants
Source: PLoS One. 2019 Feb 11;14(2):e0211861. doi: 10.1371/journal.pone.0211861 (PMC6370187; doi:10.1371/journal.pone.0211861)
Supplement: S4 Table — (DOCX) [file pone.0211861.s004.docx]

| **Table S4** Descriptive statistics of AB/PAS-positive GCs in P1d1 subpopulation | | | |
| --- | --- | --- | --- |
|  |  | **P1d1 IL-13-** | **P1d1 IL-13+** |
| **AB/PAS (%)** | Number of values | 4 | 4 |
|  | Minimum | 22.30 | 23.20 |
|  | 25% Percentile | 23.50 | 23.28 |
|  | **Median** | **27.60** | **24.40** |
|  | 75% Percentile | 37.10 | 30.63 |
|  | Maximum | 40.10 | 32.40 |
