# Supplementary material for: Interleukin-13 maintains the stemness of conjunctival epithelial cell cultures prepared from human limbal explants
Source: PLoS One. 2019 Feb 11;14(2):e0211861. doi: 10.1371/journal.pone.0211861 (PMC6370187; doi:10.1371/journal.pone.0211861)
Supplement: S5 Table — (DOCX) [file pone.0211861.s005.docx]

| **Table S5** Descriptive statistics of total CFE | | | | | | | | | |
| --- | --- | --- | --- | --- | --- | --- | --- | --- | --- |
|  |  | **P0 IL-13-** | **P0 IL-13+** | **P1 IL-13-** | **P1 IL-13+** | **P2 IL-13-** | **P2 IL-13+** | **P1d1 IL-13-** | **P1d1 IL-13+** |
| **Total CFE (%)** | Number of values | 6 | 6 | 6 | 6 | 6 | 6 | 5 | 5 |
|  | Minimum | 0.60 | 5.60 | 0.20 | 0.70 | 0.00 | 0.00 | 0.00 | 0.00 |
|  | 25% Percentile | 0.60 | 7.18 | 0.28 | 0.93 | 0.00 | 0.08 | 0.05 | 0.05 |
|  | **Median** | **1.05** | **8.15** | **0.55** | **2.00** | **0.10** | **0.10** | **0.20** | **0.10** |
|  | 75% Percentile | 1.75 | 12.85 | 1.23 | 2.68 | 0.20 | 0.23 | 0.20 | 0.20 |
|  | Maximum | 2.20 | 13.90 | 1.30 | 3.50 | 0.50 | 0.30 | 0.20 | 0.20 |
